# Clinical Controversy Surrounding the Differential Diagnosis of Branchiogenic Carcinoma

**DOI:** 10.1155/2022/4582262

**Published:** 2022-09-16

**Authors:** Alexander Karatzanis, Kleanthi Mylopotamitaki, Eleni Lagoudaki, Emmanuel Prokopakis, Sofia Agelaki

**Affiliations:** ^1^Department of Otorhinolaryngology-Head and Neck Surgery, School of Medicine, University of Crete, Heraklion, Greece; ^2^Department of Otorhinolaryngology-Head and Neck Surgery, General Hospital of Heraklion “Venizeleio-Pananeio,”, Heraklion, Crete, Greece; ^3^School of Medicine, University of Crete, Heraklion, Greece; ^4^Department of Pathology, University General Hospital of Heraklion, Heraklion, Greece; ^5^Department of Medical Oncology, University General Hospital of Heraklion, Heraklion, Greece; ^6^Laboratory of Translational Oncology, School of Medicine, University of Crete, Heraklion, Greece

## Abstract

Clinical evaluation, differential diagnosis, and management of a neck mass constitute commonly encountered problems for the head and neck surgeon. An asymptomatic neck mass in adults may be the only clinical sign of head and neck cancer. A 50-year-old female patient presented with a painless, slowly enlarging, left lateral neck lump. Ultrasonography described a possible lymph node with cystic degeneration, and fine needle aspiration biopsy only detected atypical cells of squamous epithelium. An open biopsy under general anesthesia was performed. Histopathological findings suggested the diagnosis of lymph node infiltration by squamous cell carcinoma of an unknown primary site, but differential diagnosis also included branchiogenic carcinoma arising in a branchial cleft cyst. A diagnostic algorithm for metastatic squamous cell carcinoma of an unknown primary site was followed, including positron emission tomography with computed tomography. The patient underwent panendoscopy and bilateral tonsillectomy, and an ipsilateral p16 positive tonsillar squamous cell carcinoma was detected. Further appropriate management followed. The existence of true branchiogenic carcinoma is controversial. When such a diagnosis is contemplated, every effort should be made to detect a possible primary site. Branchiogenic carcinoma, if exists at all, remains a diagnosis of exclusion.

## 1. Introduction

A neck mass is defined as a congenital or acquired abnormal lesion that is visible, palpable, or seen in an imaging study and is a frequently encountered clinical problem [1–3]. The clinical characteristics, location, and distinction between solid and cystic or cyst-like lesions assist in the diagnostic process [4]. Inflammatory and neoplastic diseases constitute the majority of cystic or cyst-like neck masses in adults [2]. Etiologies may be grouped according to whether the onset or duration is acute (e.g., infectious), subacute (e.g., squamous cell carcinoma), or chronic (e.g., bronchocele), and further narrowed by patient demographics [5]. An asymptomatic neck mass may be the initial or only clinically apparent manifestation of head and neck cancer [6]. Thus, persistent asymptomatic neck masses in an adult should be considered malignant until proven otherwise [3].

Branchial cleft cyst carcinoma is the result of malignant squamous cell degeneration of branchial remnants [7]. A strong debate exists regarding the differential diagnosis between primary branchial cleft cyst carcinoma and cystic cervical deposits of metastatic squamous cell carcinoma of the upper aerodigestive tract [8, 9]. Many authors claim that most cases of branchial cleft cyst carcinoma reported in the literature are secondary metastatic lesions from an occult carcinoma in the Waldeyer's ring [10, 11]. Considering the benign nature of the second arch branchial cleft cyst, primary malignant tumors that originate from the cyst should be quite rare [12]. Branchiogenic carcinoma is, at best, a diagnosis of exclusion [13]. Consequently, all patients over the age of 40 years who present with malignant cervical masses, arising in anatomical regions where branchial cleft anomalies are developed, should first be assumed to have occult primary tumors [11, 14]. In this report, we present a case of a neck mass in an adult where the original biopsy presented a diagnostic dilemma between branchiogenic carcinoma and metastatic squamous cell carcinoma of an unknown primary site. Subsequent diagnostic workup and surgery revealed the presence of metastatic p16^+^ tonsillar carcinoma.

## 2. Case Report

A 50-year-old female presented at a tertiary referral center with a painless, slowly enlarging, left lateral neck lump that had been noticed for nearly four weeks. Based on medical history, she was otherwise healthy, aside from smoking, 30 pack-years, hypercholesterolemia, and allergy to citrus fruits. Otolaryngologic examination revealed a well-circumscribed mass located in the left level II of the neck. On palpation, the mass was rubbery, well-circumscribed, nontender, and mobile. No other significant findings were found on palpation. The rest of the head and neck examination, including flexible upper airway endoscopy, was normal. Antibiotic treatment was prescribed for a short period and ultrasound imaging was recommended.

Ultrasonography of the neck described an enlarged, oval lymph node on the left jugulo-digastric angle, with a longitudinal axis of 2.95 cm and a short axis of 1.85 cm. The lymph node was characterized by cystic degeneration, with hyperechogenic elements, and no significant vessel flow. It had sharp margins with central parenchymal anechoic regions. The absence of extranodal extension or parenchymal calcification was noted. An avascular appearance, with no visible hilum, was also identified. The border between the cortex and medulla was indefinite.

**Figure 1:**
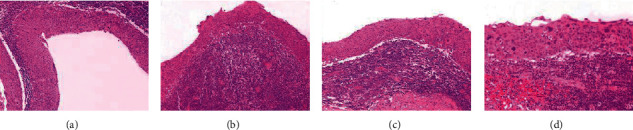
Histopathologic examination of the excised cervical lymph node revealed multiple cystic epithelial structures lined by stratified squamous epithelium exhibiting to a great extent malignant cytologic (hyperchromatic, irregular nuclei, mitotic figures) and architectural (loss of polarity) features (a); lying on dense lymphatic tissue focally forming lymphoid follicles with prominent germinal centers (b). The squamous cell epithelium lining of the cysts exhibited areas of transition (b, c) of moderate/severe dysplasia to in situ squamous cell carcinoma (d). A well-defined sinus tract was not present. Hematoxylin and eosin-stained slides of the cervical lymph node (a–d) at X40 (a, b, and c); and x200 (d) magnification.

A decision to perform fine needle aspiration biopsy was taken. The lymph node was aspirated with ultrasound-guided fine-needle aspiration. Cytology revealed atypical cells of squamous epithelium with no additional information. Consequently, an open biopsy under general anesthesia was performed. The mass in the left level II of the neck was dissected and excised through a relevant 3 cm transverse cervical skin incision. Interestingly, macroscopic intraoperative evaluation, including position and consistency of the mass, was highly suggestive of a branchial cleft cyst of the second branchial arch. The postoperative course was uneventful. Histologic examination described a solid white-brown neoplastic mass, measuring 4.1 × 2.4 × 1.8 cm, with intranodal focal cystic lesions lined with keratinizing squamous cell carcinoma. Immunohistochemistry was positive for KYMB and p63, but negative for p16 (Figure 1). The histopathological findings suggested that the most likely diagnosis was lymph node infiltration by squamous cell carcinoma of an unknown primary site. Differential diagnosis also included a branchiogenic carcinoma arising in a branchial cleft cyst.

A diagnostic algorithm for metastatic squamous cell carcinoma of an unknown primary site was initiated. Magnetic resonance imaging with intravenous paramagnetic contrast agent injection only depicted a lymph node with a transverse diameter of 1.1 cm posterior to the right submandibular salivary gland. Symmetrical swelling of the palatine tonsils without focal lesions was observed. Positron emission tomography with computed tomography, with intravenous 353 MBq 18-fluorodeoxyglucose administration, showed lymph nodes in the left level II as foci of hypermetabolism, suspicious for malignancy. It also displayed a lymph node with a short axis of 7 mm in the left level IV (SUVmax: 3.4 and 3.2). Moreover, diffuse symmetrical hypermetabolism in the palatine and lingual tonsils was noted.

**Figure 2:**
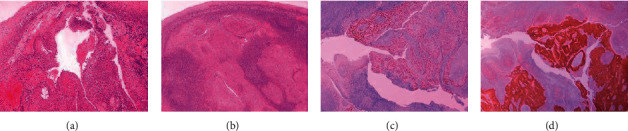
Crypt of the left palatine tonsil lined by squamous cell epithelium lining showing areas of dysplasia and progression (a) to infiltrative moderately differentiated keratinizing carcinoma in the form of solid coalescent aggregations with foci of keratinization and comedo necrosis neoplastic squamous cells with frank atypia (b). Immunohistochemically, the neoplastic cells were positive for p63 (c) and showed strong and diffuse positivity for p16 (d). Hematoxylin and eosin-stained slides of the left palatine tonsil (a–d) at x20 (b, c, and d) and x40 (a) magnification.

The patient underwent panendoscopy under general anesthesia, where a bilateral tonsillectomy was performed, along with biopsies from the base of the tongue and nasopharynx. In addition, and in accordance with the findings of positron emission tomography with computed tomography, a functional neck dissection of the left side was performed. The postoperative course was uneventful. Permanent histopathology of the left palatine tonsil, 3 × 1.8 × 1.2 cm in size, revealed a focus of moderately differentiated, squamous cell carcinoma measuring 0.9 cm. Ιmmunohistochemistry was p16+, p53+ in 2–3%, and PD-L1+ localized in 2–3% (Figure 2). All other tissue specimens, including lymph nodes from the neck dissection, were negative for malignancy.

The patient was diagnosed with a human papillomavirus (HPV) positive tonsillar squamous cell carcinoma, pT1N1M0, stage I, according to the American Joint Committee on Cancer, eighth edition [15]. The tumor board indicated the need for adjuvant treatment. This consisted of intensity-modulated radiotherapy of the primary site and regional cervical lymph nodes (70 Gy and 50 Gy, respectively) administered in eight weeks, with three cycles of platinum-based concurrent chemotherapy (cisplatin 100 mg/m2), administered intravenously every three weeks. Due to sensorineural hearing loss and grade II allergic reaction to platinum in the first administration, chemotherapy was discontinued. The patient continued with concurrent radiotherapy and cetuximab administered as a 400 mg/m2 initial dose, followed by 250 mg/m2 intravenously weekly. The patient completed treatment and has now been placed on regular follow-up. One year later, there was no evidence of recurrence.

## 3. Discussion

A neck mass may pose a diagnostic dilemma for the head and neck surgeon, and the underlying cause may not always be identified. Neck masses are classified as masses below the mandible, above the clavicle, and deep to the skin, although they may secondarily involve the overlying skin [3]. The differential diagnosis for a neck mass is quite extensive [16]. Etiologies may be grouped according to whether the onset or duration is acute (e.g., infectious), subacute (e.g., squamous cell carcinoma of the upper aerodigestive tract), or chronic (e.g., branchial cleft cyst or thyroid pathology), and further narrowed by patient demographics [5]. Acute masses appear over a short period and are generally symptomatic [5]. Subacute masses are noticed within weeks to months, and chronic masses are usually evident as long-standing [5]. The patient's age is very important for guiding diagnosis. Infections cause most of the neck masses in children. For patients younger than 40 years, initial considerations should focus on congenital anomalies, such as branchial cleft cyst, and infectious/inflammatory causes [16]. For patients older than forty years, 80% of neck masses are subacute, associated with a neoplastic process [16]. Such a neck mass should therefore be considered malignant until proven otherwise [3].

The role of human papillomavirus in carcinogenicity is known for decades [17]. In 2007, HPV was recognized as a risk factor for oropharyngeal squamous cell carcinoma [18]. High-risk HPV is classified as the most common sexually transmitted infection in the United States, and both oral and genital HPV transmission is associated with sexual activity [19]. According to epidemiological data, HPV-positive oropharyngeal squamous cell carcinoma increased by 225% from 1988 to 2004 [20]. Patients with HPV-positive oropharyngeal squamous cell carcinoma are younger, more likely to be white, with fewer smoking pack-years, smaller primary tumors, and significantly better outcomes, with higher overall survival rates [21].

A cystic mass in the head and neck may be a primary presentation of occult malignancy. An overall malignancy rate of 10.7–14.4% was reported in patients initially diagnosed with a lateral neck cyst [22, 23]. Patients with HPV-positive oropharyngeal squamous cell carcinoma are likely to present with an asymptomatic neck mass, and the cervical lymph nodes are frequently cystic [24]. These are often misdiagnosed as branchial cleft cysts, leading to delayed diagnosis and delayed treatment [25]. They tend to have large nodal involvement and early tumor stage, so the majority of patients with HPV-positive head and neck squamous cell carcinoma are diagnosed at clinically advanced stages [26]. In 2017, the American Joint Committee on Cancer and the Union for International Cancer Control introduced a separate staging system for patients with HPV-positive oropharyngeal squamous cell carcinoma [15].

Second arch branchial cleft cysts or branchiomas related to the second pouch are the most common type of branchial anomalies [27, 28]. They represent approximately 22% of congenital neck masses [29]. Branchiogenic carcinoma is thought to be the result of malignant squamous cell degeneration, within the confines of epithelial remnants derived from the embryonic branchial apparatus [7]. The existence of a primary carcinoma arising from a branchial cleft cyst was first proposed by Volkmann in 1882 [8, 9]. He was the first to introduce the concept of “branchiogenic carcinoma” [9]. In 1950, Martin et al. reviewed fewer than 40 cases and initially recommended standard criteria for establishing the diagnosis of branchial cleft cyst carcinoma. In 1989, Khafif et al. established stricter criteria for the definitive diagnosis of branchiogenic carcinoma. These include (1) the location of the tumor to be in the anatomic region of the branchial cleft cyst or sinus, (2) the histologic appearance of the tumor consistent with its origin from branchial vestiges, e.g., squamous cell carcinoma, (3) the presence of the carcinoma within the lining of an identifiable epithelial cyst, (4) the identification of transition from the normal squamous epithelium of the cyst to carcinoma and, (5) the absence of any identifiable primary malignant tumor after an exhaustive evaluation of the patient, including endoscopy, complete CT scan of the head and neck and suitable biopsy [8, 27].

Typical branchiogenic carcinoma clinical presentation is characterized by a benign cystic mass in the upper lateral neck, often indistinguishable from a branchial cleft cyst, during the fifth or sixth decade of life [8]. The duration of symptoms may range from 2 weeks to 40 years, with no correlation of survival with the duration of the existence of the branchial cleft cyst, suggesting that the carcinoma is possibly developing within a long-standing benign branchial cyst [8]. However, the natural behavior of branchial cleft cyst carcinoma cannot be predicted from the few cases previously reported [8]. In some cases, the tumor seems to be aggressive with a significant incidence of local recurrence, multiple nodal metastases, often bilateral, and poor outcome [8, 30]. The extent of the disease is correlated to the prognosis [31]. Relevant literature suggests that the therapeutic approach must be aggressive including wide local excision, neck dissection, and adjuvant radiotherapy and/or chemotherapy, when an invasion of surrounding tissue or cervical lymph nodes is present, in order to prevent a recurrence [31].

There is a contemporary debate regarding the differential diagnosis between primary branchiogenic carcinoma and cystic cervical deposits of metastatic squamous cell carcinoma. Some authors claim that cystic cervical deposits of squamous cell carcinoma will ultimately prove to be of metastatic origin, even in the absence of a detectable primary tumor. [13]. They assert that the original diagnostic criteria for branchiogenic carcinoma were incomplete, due to the absence of panendoscopy and tonsillar biopsy, limited follow-up in many cases, and typical administration of radiation therapy, which might have eradicated occult primary tumors and so created the impression of a true primary cervical neoplasm [32, 33]. Due to the shared anatomic region of a branchial cleft cyst carcinoma with a metastatic occult primary of the upper aerodigestive tract, and the difficulty of histologically distinguishing a branchial cleft cyst carcinoma from the cystic degeneration of a metastatic lymph node, there still exist barriers for making the proper distinction in every case [34].

Thompson and Heffner in their review of 136 cases, did not find one case of true branchiogenic carcinoma. They concluded that almost all presumed branchiogenic carcinomas are actually metastatic primaries from the Waldeyer's ring, and these carcinomas are clinically distinct subsets that behave differently than most oropharyngeal squamous cell carcinomas [10]. Soh suggested that most cases of branchiogenic carcinomas reported in the literature may have been secondary metastatic lesions from an occult tonsillar carcinoma [11]. A case report by McLean presented a patient with concurrent oropharyngeal squamous cell carcinoma and branchial cleft cyst that were positive for P16 and HPV DNA [35]. Lack of evidence of carcinogenic change of the normal epithelium and an identifiable normal lymphoepithelial lining of a cystic space would fail to prove that the tumor originated from the epithelium, and fail to differentiate it from a central necrotic metastatic lymph node [7]. All these data suggest that branchiogenic carcinoma is at best a diagnosis of exclusion [13]. Every adult patient over the age of 40 years presenting with a malignant cervical mass, arising in anatomical regions where branchial cleft anomalies develop, should be assumed to have an occult primary tumor [11, 14]. A diagnosis of primary branchiogenic carcinoma should be viewed with a serious dose of skepticism.

The definite diagnosis and treatment, based on differentiating between the branchial cleft cyst carcinoma or the metastasis of another primary cancer into a lymph node, are critical for patients who present with a lateral neck mass. According to guidelines for the evaluation of a neck mass in adults, thorough history and a complete head and neck examination are crucial in the initial workup [3]. A targeted physical examination should include visualizing the mucosa of the larynx, base of the tongue, and pharynx [3]. The ability to place pathology within a neck space is the first step to generating a differential diagnosis [17]. During the physical examination of a neck mass, the clinician should seek to determine the location, size, and character of the lesion [2]. Furthermore, it should be identified whether the mass is tender to palpation, pulsating, and fixated to the overlying skin or underlying structures [2]. In addition, the patterns of head and neck lymph drainage may offer clues for finding a primary source in a patient presenting with a neck mass [2]. Clinicians should prescribe antibiotic therapy for patients with a neck mass only if there are signs and symptoms of bacterial infection, such as unilateral cervical adenopathy or fever. Fine-needle aspiration is strongly recommended initially instead of open biopsy [3, 25]. Contrast-enhanced computed tomography (or magnetic resonance imaging) of the neck should be considered a first-line diagnostic investigation for all patients at increased risk of malignancy [3].

If there are no clues from the above, an open biopsy is an ultimate tool to diagnose a neck mass [3]. Open excisional biopsy of the lateral neck mass is recommended to establish a definitive diagnosis and is preferred, especially about cystic masses, to reduce the risk of tumor spillage into the wound [3]. Ancillary tests should also be performed and may include complete blood count, estimated sedimentation rate, thyroid ultrasound, computed tomography of the chest with contrast, and specific infection tests (e.g., HIV, Epstein–Barr virus, cytomegalovirus, and tuberculosis) (3,29). Positron emission tomography with computed tomography using fluorodeoxyglucose tracer is also recommended after the diagnosis of metastatic squamous cell carcinoma of an unknown primary site, and before panendoscopy so that any suspicious areas of increased uptake can be biopsied [25]. Positron emission tomography with computed tomography has significant sensitivity and specificity in identifying occult primary lesions, as well as in assessing recurrent head and neck tumors [3]. Finally, panendoscopy and examination of the upper aerodigestive tract under general anesthesia with biopsies are strongly recommended in order to reveal a primary tumor site as the source of metastatic spread to a regional lymph node [3].

## 4. Conclusion

The incidence of branchiogenic carcinoma is significantly lower compared to metastatic neck nodes. Even when, according to Khafif's criteria, a histopathologic diagnosis of branchial cleft cyst carcinoma may correctly be established, clinicians should always care to exclude the presence of an occult primary tumor. It should be further kept in mind that neck metastases from carcinomas arising in the Waldeyer's ring, especially cervical metastases from HPV-positive oropharyngeal squamous cell carcinoma, are cystic, and a minority of malignant cystic neck masses may present without an obvious primary tumor.
